# Absence of dynamic strain aging in an additively manufactured nickel-base superalloy

**DOI:** 10.1038/s41467-018-04473-5

**Published:** 2018-05-25

**Authors:** Allison M. Beese, Zhuqing Wang, Alexandru D. Stoica, Dong Ma

**Affiliations:** 1 Department of Materials Science and Engineering, Pennsylvania State University, University Park, PA 16802 USA; 2 Department of Mechanical Engineering, Pennsylvania State University, University Park, PA 16802 USA; 3 Neutron Scattering Division, Spallation Neutron Source, Oak Ridge National Laboratory, Oak Ridge, TN 37831 USA

## Abstract

Dynamic strain aging (DSA), observed macroscopically as serrated plastic flow, has long been seen in nickel-base superalloys when plastically deformed at elevated temperatures. Here we report the absence of DSA in Inconel 625 made by additive manufacturing (AM) at temperatures and strain rates where DSA is present in its conventionally processed counterpart. This absence is attributed to the unique AM microstructure of finely dispersed secondary phases (carbides, N-rich phases, and Laves phase) and textured grains. Based on experimental observations, we propose a dislocation-arrest model to elucidate the criterion for DSA to occur or to be absent as a competition between dislocation pipe diffusion and carbide–carbon reactions. With in situ neutron diffraction studies of lattice strain evolution, our findings provide a new perspective for mesoscale understanding of dislocation–solute interactions and their impact on work-hardening behaviors in high-temperature alloys, and have important implications for tailoring thermomechanical properties by microstructure control via AM.

## Abstract (web summary)

Detrimental serrated plastic flow via dynamic strain aging (DSA) in conventionally processed nickel superalloys usually occurs during high temperature deformation. Here, the authors suppress DSA via a unique microstructure obtained using additive manufacturing and propose a new dislocation-arrest model in nickel superalloys.

## Introduction

Dynamic strain aging (DSA) is ubiquitous in metal alloys, including Al, Cu, Fe, and Ni-base alloys, when they are plastically deformed within specific ranges of temperature and strain rate^1–6^. It is conventionally believed that DSA occurs due to the interaction between solute atmospheres that build up around dislocations as they move through the matrix and secondary phases within the matrix^1,7–12^. On the macroscale, DSA leads to discernible serrations in the stress–strain curve. Many adverse materials properties are associated with DSA, including reduction in fracture resistance in Al–Li alloys^13^ and decrease in low-cycle fatigue life and loss of ductility in stainless steels^14–16^ and nickel-base superalloys^16–18^. Thus, understanding the mechanisms of DSA is of great scientific and technological importance. However, despite extensive studies on DSA since the 1950s, the underlying physics of DSA are still not clear, with seemingly inconsistent explanations in the literature.

In this study, we provide a new perspective for understanding, at the mesoscale, dislocation–solute interactions that dictate DSA and their impact on work-hardening behavior. We first demonstrate that DSA is absent in an additively manufactured nickel-base superalloy, Inconel 625 (IN625), when deformed at elevated temperatures. This contrasts with the presence of DSA in its conventionally processed IN625 counterpart. With in situ neutron diffraction studies of lattice strain evolution, we show how the initiation of DSA-induced serrations relates to grain orientation. Based on these observations and those of the unique AM microstructure, specifically with respect to texture and secondary phase formation, we propose a dislocation-arrest model to rationalize the criterion for the onset and absence of DSA in Ni-base alloys. With the rapid rise of AM as a means for fabricating complex components, particularly out of difficult-to-machine materials such as Inconel 625, an understanding of the structure–property relationships is paramount for the adoption of these components in structural applications. This work also has implications for tailoring the microstructure in additively manufactured components to improve properties, specifically, by eliminating DSA at elevated temperatures at which Inconel 625 is often used.

## Results

### Fabrication by additive manufacturing

The IN625 made by AM (AM-IN625) was deposited using laser-based directed energy deposition (DED), in which a laser melts a metallic substrate material, and pre-alloyed metallic powder feedstock is delivered to the melt pool via a nozzle^19–23^. As the laser advances, the material cools, solidifies, and fuses to the layer below. As each subsequent layer is deposited, the material in the component undergoes rapid solidification and thermal cycles.

### In situ neutron diffraction characterization upon loading

**Fig. 1:**
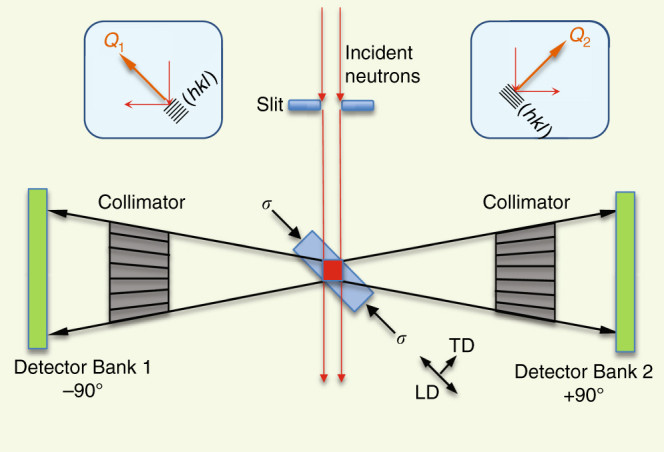
Schematic of in situ neutron diffraction experimental set-up on VULCAN. Top down view, not to scale. The red square represents the sampling volume defined by the incident beam and the radial collimators. The −90° and +90° detector banks record diffractions from the grains whose lattice planes (*hkl*) are perpendicular to *Q*_1_ and *Q*_2_, respectively, as shown in the two insets. The compression specimen (light-blue rectangle) is positioned at 45° from the incident beam such that Bank 1 probes the strain component along the loading direction (LD) while Bank 2 simultaneously detects the strain component in the transverse direction (TD)

Uniaxial compression tests at room temperature, 600 °C, and 700 °C, along with in situ neutron diffraction characterization (see Methods, Supplementary Notes 1 and 2, and Supplementary Table 1), were performed at Oak Ridge National Laboratory’s Spallation Neutron Source using the VULCAN instrument as schematically shown in Fig. 1^24^. Cylindrical specimens (5 mm diameter and 10 mm long) were extracted from the AM-IN625 along the build length direction as well as from conventionally processed IN625 (CP-IN625) plate^25^. Equipped with two detector banks positioned at −/+90° diffraction angles, VULCAN allows for simultaneous measurement of two diffraction patterns corresponding to two orthogonal scattering vectors, i.e., along the loading and transverse directions (LD and TD, respectively)^26^. In situ neutron diffraction was used to measure the *hkl*-dependent lattice strains during loading, i.e., $$\varepsilon _{hkl} = \frac{{d_{hkl} - d_{hkl}^0}}{{d_{hkl}^0}},$$ where *d*_*hkl*_ is the measured *hkl*-specific interplanar spacing during deformation and $$d_{hkl}^0 $$ is the stress-free reference interplanar spacing at each temperature.

### Macroscopic mechanical properties

**Fig. 2:**
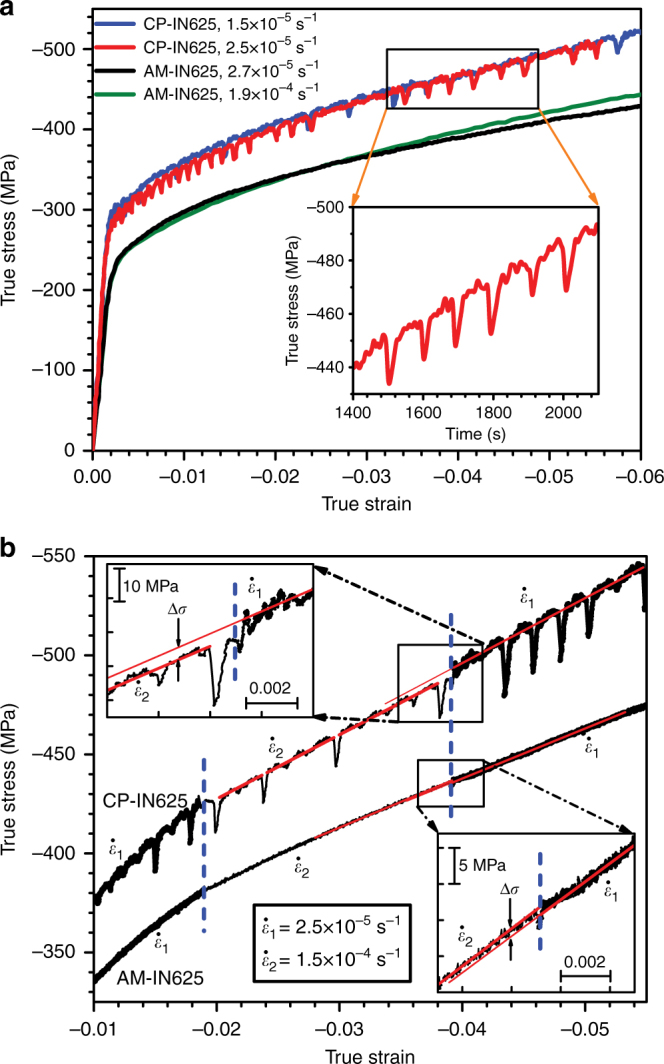
High temperature mechanical behavior of conventionally processed Inconel 625 (CP-IN625) and additively manufactured Inconel 625 (AM-IN625). **a** Macroscopic true stress–strain curves for CP-IN625 and AM-IN625 samples deformed at different strain rates at 600 °C. The inset shows serrations in a corresponding true stress versus time plot. **b** True stress–strain curves for strain-rate change tests of AM-IN625 and CP-IN625 at 600 °C. Insets in upper left and lower right are the enlarged views of the portion where the second strain-rate change takes place, showing how the stress change (Δ*σ*) is determined

The stress–strain curves of CP-IN625 and AM-IN625 were smooth under constant strain rate loading at room temperature (see Supplementary Fig. 3). At 600 °C (and strain rates ranging from 1.5 × 10^−5^ s^−1^ to 1.9 × 10^−4^ s^−1^), the stress–strain curves for the CP-IN625 exhibited serrations indicative of DSA, while the AM-IN625 did not exhibit any serrations, as shown in Fig. 2. The presence of serrations in the CP-IN625 and absence of serrations in AM-IN625 was also observed during deformation at 700 °C at strain rates on the order of 1.5 × 10^−5^ s^−1^ (see Supplementary Fig. 4). Additionally, at each of the three temperatures studied, the yield strength and flow stress as a function of plastic strain were higher in the CP-IN625 compared to AM-IN625.

The serrations observed for the CP-IN625 are classified as Type C serrations, in which the absolute value of the stress drops below that of the continuous stress–strain curve during deformation. At 600 °C, and a strain rate of 2.5 × 10^−5^ s^−1^, the flow stress dropped about 15 MPa below the overall flow stress approximately every 100 s, as illustrated in the inset of Fig. 2a. Comparing the CP-IN625 constant strain rate tests at the two strain rates studied, DSA was discernible at both strain rates as shown in Fig. 2a; however, the 0.2% offset yield strength of the sample deformed at 2.5 × 10^−5^ s^−1^ was lower than that of the sample deformed at a slower strain rate of 1.5 × 10^−5^ s^−1^, indicating a decrease in stress of ∼10 MPa due to an increase in strain rate of 1.0 × 10^−5^ s^−1^. For the AM-IN625, the sample subjected to a strain rate of 1.9 × 10^−4^ s^−1^, seven times that of the sample deformed at 2.7 × 10^−5^ s^−1^, had essentially the same 0.2% offset yield strength as that in the lower strain rate test, and neither of their stress–strain curves showed discernible serrations.

In order to determine the strain-rate sensitivity (*γ*), we carried out strain-rate change tests on CP-IN625 and AM-IN625 at 600 °C, as shown in Fig. 2b. Following Mulford and Kocks^9^ and van den Brink et al.^27^, we define $\gamma = \Delta \sigma /\Delta {\mathrm{ln}}\dot {\varepsilon}$, where $\dot \varepsilon$ is the strain rate and Δ*σ* is the change in flow stress extrapolated from the post-transition portion of the flow stress curve to the strain at which the strain-rate change was made, as shown in the upper left and lower right insets in Fig. 2b. As such, *γ* was determined to be approximately +0.5 MPa·s for AM-IN625, and approximately −2.2 MPa·s for CP-IN625. It is noted that the negative value of *γ* for the CP-IN625 is comparable to the values observed for Inconel 600 (~−2.0 to −3.0 MPa·s) at 437 °C under similar stresses^9^. Additional tests performed on as-deposited and heat-treated AM-IN625 samples are presented in Supplementary Note 2.

All the mechanical tests at 600 °C demonstrate that CP-IN625 exhibits a negative strain-rate sensitivity while AM-IN625 shows a positive strain-rate sensitivity. This explains why DSA is present in the CP-IN625 but is absent in AM-IN625, as will be discussed later.

### Microstructure

To explain the presence of DSA in CP-IN625 and the absence of this phenomenon in AM-IN625, we examined the microstructure, composition, and grain orientation-dependent stress–strain behavior. Inert gas fusion and inductively coupled plasma optical emission spectroscopy revealed no significant difference in the chemical compositions of the samples as shown in Supplementary Table 3.

**Fig. 3:**
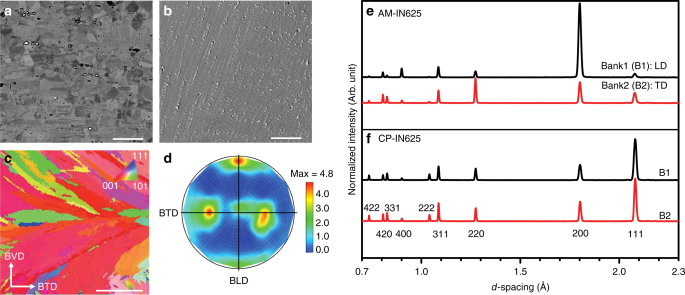
Microstructure and texture in conventionally processed and additively manufactured Inconel 625. **a** Backscattered electron image of etched CP-IN625 with Mo- and Nb-rich (bright) and Ti-rich (dark) carbides. Scale bar: 50 μm. **b** Secondary electron image of etched AM-IN625 with Mo- and Nb-rich carbides, N-rich phase, and Laves phase. Scale bar: 50 μm. **c** EBSD map of planes whose normal is parallel to the build length direction (BLD). Here BTD denotes the build thickness direction, and BVD denotes the build vertical direction. The colors correspond to the *hkl* plane normals coming out of the page. Scale bar: 500 μm. **d** (200) pole figure of the AM build determined from neutron diffraction, showing a Goss texture component: {011} <001>. The center of the pole figure is parallel to the BVD. **e**, **f** Neutron diffraction patterns, in the loading direction (LD) and transverse direction (TD), of AM-IN625 and CP-IN625 samples at 600 °C prior to loading

The CP-IN625 exhibited an equiaxed microstructure with carbides, while AM-IN625 showed a dendritic structure with carbides, N-rich phases, and interdendritic Laves phase (see Fig. 3a, b, Supplementary Figs. 8 and 10-13, and Supplementary Tables 5-7 in Supplementary Note 3). The area fraction of secondary phases in Fig. 3a, b was found to be approximately 1% in the CP-IN625 and 2% in AM-IN625. However, these secondary phases in the AM-IN625 are 0.3–1.3 µm in diameter, thus finer and more closely spaced than the carbides in the CP-IN625, which have diameters of 0.5–8 µm. Energy-dispersive X-ray spectroscopic analysis indicated that the carbides and Laves phase in the AM-IN625 are rich in Mo and Nb. This is consistent with literature showing that, during rapid solidification of IN625, Nb, Mo, C, and Ti segregate into the interdendritic regions, resulting in the formation of NbC carbides and Laves phase^28–31^.

Electron backscatter diffraction (EBSD) was performed to reveal the grain structure of CP-IN625 and AM-IN625, as shown in Fig. 3c, Supplementary Figure 9, and Supplementary Table 4. A representative EBSD color map for AM-IN625 in Fig. 3c indicates a strong texture of the <100> direction along the build length (the compression axis of the samples). This agrees with the pole figures (Fig. 3d and Supplementary Fig. 2) determined by neutron diffraction, indicative of a Goss texture component {011}<001> (see Supplementary Note 1). Typical neutron diffraction patterns for as-deposited AM-IN625 and CP-IN625 are given in Fig. 3e, f.

### DSA models

**Fig. 4:**
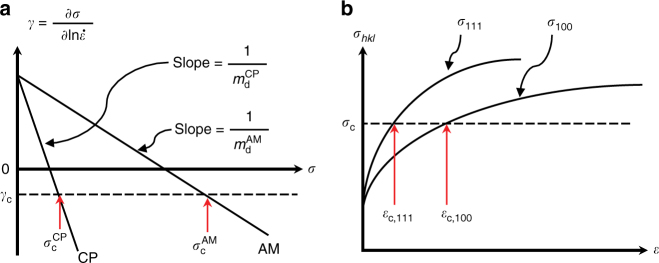
Theoretical strain-rate sensitivity and strain-hardening behavior of Inconel 625. **a** Schematic of strain-rate sensitivity as a function of flow stress for conventionally processed and additively manufactured Inconel 625 at a given temperature, where *γ*_c_ (<0) denotes the critical value of *γ* for the onset of serrations, and $\sigma _{\mathrm{c}}^{\mathrm{CP}}$and $\sigma _{\mathrm{c}}^{\mathrm{AM}}$ are the corresponding critical stresses for initiating serrated flow for CP-IN625 and AM-IN625, respectively. **b** Schematic of *hkl*-dependent strain-hardening behaviors showing that for a given grain-level critical stress for serrations, due to their higher strain hardening rate, <111>//LD grains would reach the critical stress at a lower strain than <100>//LD grains, which have a lower strain hardening rate

With these data showing DSA in CP-IN625 and the absence of DSA in AM-IN625, prior DSA models are revisited, and a new model for DSA is proposed. It is well established that a negative value of the strain-rate sensitivity, *γ*, of the flow stress is correlated with the occurrence of macroscopic jerky flow or serrations in the stress–strain curve^9,11,27,32,33^. Therefore, a critical strain, $\varepsilon _{\mathrm{c}}$, or correspondingly a critical stress, *σ*_c_, is required to produce a critical negative value of the strain-rate sensitivity (*γ*_c_ < 0) to initiate serrations^27^. The strain-rate sensitivity of the flow stress at a temperature *T* is given by^9^:

1 $$\gamma = \frac{{\partial \sigma }}{{\partial {\mathrm{ln}}\dot \varepsilon }}\Bigg|_T = \sigma _{\mathrm{f}}\left( {\frac{1}{{m_{\mathrm{f}}}} - \frac{1}{{m_{\mathrm{d}}}}} \right) + \sigma \cdot \frac{1}{{m_{\mathrm{d}}}}$$

where $\frac{1}{{m_{\mathrm{f}}}} =$ $\frac{{\partial {\mathrm{ln}}\sigma _{\mathrm{f}}}}{{\partial {\mathrm{ln}}\dot \varepsilon }}\Bigg|_T$, $\frac{1}{{m_{\rm d}}} =$ $\frac{{\partial {\mathrm{ln}}\sigma _{\mathrm{d}}}}{{\partial {\mathrm{ln}}\dot \varepsilon }}\Bigg|_T$, *σ* ($= \sigma _{\mathrm{f}} + \sigma _{\mathrm{d}} \, > \, 0$) is the flow stress, *σ*_f_ is the flow stress component related to friction imposed by the solutes on mobile dislocations, *σ*_d_ is the flow stress component related to the dislocation–dislocation interactions that cause strain hardening, *ε* ( > 0) is the applied strain, and $\dot \varepsilon$ is the strain rate. Therefore, $\frac{1}{{m_{\mathrm{d}}}}$ is the slope of the strain-rate sensitivity versus flow stress curve. By setting $\gamma \le \gamma _{\mathrm{c}}$ < 0, as illustrated in Fig. 4a, the critical stress, *σ*_c_, for serrations to occur is given by:

2 $$\sigma _{\mathrm{c}} = m_{\mathrm{d}} \cdot \left( {\gamma _{\mathrm{c}} - \frac{{\sigma _{\mathrm{f}}}}{{m_{\mathrm{f}}}}} \right){ + } \, \sigma _{\mathrm{f}}$$

and the critical strain, $\varepsilon _{\mathrm{c}}$, can be written in terms of *σ*_c_ using the Ramberg–Osgood equation^34^:

3 $$\varepsilon _{\mathrm{c}} = \frac{{\sigma _{\mathrm{c}}}}{E}\left\{ {1 + \alpha \cdot \left( {\frac{{\sigma _{\mathrm{c}}}}{{\sigma _0}}} \right)^{n - 1}} \right\}$$

where *E* is Young’s modulus, *α* is a material constant, *σ*_0_ is the nominal yield stress, and *n* is an inverse strain-hardening exponent. The strain-rate sensitivity of the flow stress component related to friction, $m_{\mathrm{f}}$, is positive for all temperatures and strains such that the total rate sensitivity (*γ*) is positive at the beginning of plastic deformation^9^. Therefore, for *γ*_c_ < 0, $\gamma _{\mathrm{c}} - \sigma _{\mathrm{f}}/m_{\mathrm{f}}$ is always negative, and a critical strain ($\varepsilon _{\mathrm{c}} \, > \, 0$) is operative only if $m_{\mathrm{d}}$ is negative. Mulford and Kocks^9^ proposed that a negative value of $m_{\mathrm{d}}$ is due to the operation of the DSA mechanism, in which solutes interact with dislocations, affecting the work-hardening process, as $m_{\mathrm{d}}$ is negative only in the temperature range in which jerky flow occurs. Eqs. (2) and (3) indicate that the smaller the absolute value of $m_{\mathrm{d}}$, the smaller the critical strain necessary for initiating serrations. Eq. (3) also suggests that the larger the work-hardening rate (i.e., smaller *n*), the smaller the critical strain needed for a negative strain-rate sensitivity^9,11^.

### DSA model informed by microscale mechanisms

Based on the observations in the present study, and in an effort to rationalize the occurrence of Type C serrations in the CP-IN625 and their absence in the AM-IN625, we propose a model that reveals two competing effects for solute atmosphere formation on mobile dislocations, which is required for DSA. This model is based on the dislocation arrest theory originally proposed by Sleeswyk^35^ and modified by Mulford and Kocks^9^ and on the carbide–carbon reaction model proposed by Hayes and Hayes^10^. The dislocation arrest theory suggests that solute atmospheres (e.g., carbon atoms in IN625) form on forest dislocations, then drain from the forest dislocations by pipe diffusion to mobile dislocations while the mobile dislocations are temporarily arrested at the forest dislocation junctions^9,35^. In a specific temperature range, dislocation pipe diffusion is rapid enough to allow the formation of carbon atmospheres on the mobile dislocations at the forest dislocation junctions; this produces an obstacle effect with a strength that increases with waiting time, resulting in a negative $m_{\mathrm{d}}$^9^. When the flow stress exceeds the critical stress (*σ*_c_) necessary for initiating serrations (Eq. (2)), Type C serrations occur upon increased straining; the value of the stress drop reflects the increased strength due to solute–dislocation interactions during waiting time. However, for the AM-IN625 in which finer secondary phases are present and distributed mostly in the grain interiors, mobile dislocations are also arrested at these phases. The secondary phases can react with carbon and act as sinks for the carbon atmospheres, draining carbon from the mobile dislocations^10^.

Thus, for IN625, the formation of carbon atmospheres on mobile dislocations depends on two competing effects: the draining of carbon from forest dislocations via pipe diffusion onto mobile dislocations versus the sink effect of carbon being drained from mobile dislocations into carbides in the matrix. For CP-IN625, the large carbides are coarsely distributed and mostly along the grain boundaries so that the probability of arresting mobile dislocations by the carbides is low, and if non-zero, the effective contact length is rather small. Therefore, these carbides are not effective sinks of carbon atmospheres and dislocation pipe diffusion is dominant during waiting time, resulting in a negative *m*_d_ ($m_{\mathrm{d}}^{\mathrm{CP}}$ in Fig. 4a), and therefore Type C serrations upon increased straining. However, for AM-IN625, the finer, more closely spaced secondary phases, having a larger quantity, are more effective in arresting mobile dislocations with high probability and with larger contact length, such that they are effective sinks for the carbon atmospheres. This sink effect will decrease the absolute value of $1/m_{\mathrm{d}}$ ($m_{\mathrm{d}}^{\mathrm{AM}}$ in Fig. 4a), resulting in a significantly larger critical stress (Eq. (2)) or strain (Eq. (3)) for negative strain-rate sensitivity, which is experimentally undetectable.

Furthermore, if the carbides drain carbon from the mobile dislocations faster than that needed for carbon-atmosphere formation by dislocation pipe diffusion, $m_{\mathrm{d}}$ will become positive, leading to a positive strain-rate sensitivity versus stress trend, and the complete disappearance of DSA. Similarly, if forest dislocations are arrested by secondary phases that drain carbon away, carbon-atmosphere formation on forest dislocations will be impeded, resulting in a decrease in the draining of carbon from forest to mobile dislocations, reducing the propensity for DSA.

Therefore, in all of these scenarios, the DSA effect is significantly reduced by the closely spaced secondary phases, preventing the critical carbon build-up needed for dislocation locking and unlocking and making Type C serrations either experimentally undetectable or completely absent in AM-IN625. Additional tests on heat-treated AM-IN625 (see Supplementary Table 2) showed that, with partially dissolved secondary phases, DSA appeared with a low stress drop (see Supplementary Fig. 5), and with fully dissolved secondary phases, DSA appeared with a similar level stress drop as seen in CP-IN625 (see Supplementary Fig. 6). The appearance of DSA with the dissolving of secondary phases in AM-IN625 provides additional evidence of the above-reasoned microstructural mechanisms for DSA.

Previous literature on DSA has reported conflicting results in terms of whether interstitial atoms have a significant effect^1,36^ or little effect on DSA^33,37^. Therefore, the tests reported here were analyzed (see Supplementary Fig. 14) to compute the activation energy of DSA as detailed in Supplementary Note 4. The activation energy calculated (121 kJ mol^−1^) for the present experiments is similar to that of C diffusion in Ni^38^ and much lower than that of diffusion of substitutional atoms in Ni^39,40^. This indicates that the DSA under the presently studied temperature and strain-rate conditions was primarily controlled by interstitial C.

### Mesoscale mechanisms of DSA

The in situ neutron diffraction experiments reported herein reveal that crystallographic texture is also important in DSA. It has been shown in stainless steels and Inconel 600 that work-hardening rate versus temperature is parabolic, with a peak in the temperature range where Type C serrations occur^9,11^. Although Eqs. (2) and (3) give no explicit relationship between work-hardening rate and the critical strain, they imply that $\varepsilon _{\mathrm{c}}$ is associated with $m_{\mathrm{d}}$ and *n*, which are determined by the work-hardening process. This relationship indicates that a higher work-hardening rate, associated with a smaller *n* and a smaller absolute value of $m_{\mathrm{d}}$, will give rise to a smaller $\varepsilon _{\mathrm{c}}$ required to initiate serrations. At 600 °C, it is found that the work-hardening rate of the CP-IN625 is ∼27% higher than that of AM-IN625 (see Table 1). This is likely due to two effects: the lack of DSA as discussed above, and the presence of textured grains near the <100> orientation in the LD in AM-IN625 (Fig. 3c, d), as discussed below. Thus, in addition to the sink effect of the secondary phases, which increases the absolute value of the negative $m_{\mathrm{d}}$, texture softening that reduces the work-hardening rate may also contribute to the absence of Type C serrations in AM-IN625.

**Table 1:** Work-hardening rates, at 600 °C, determined experimentally for conventionally processed and additively manufactured Inconel 625 samples and their <*hkl*>-specific values

	Work-hardening rate, *θ*	
	CP-IN625	AM-IN625
Sample	31.7 ± 0.6	25.0 ± 0.3
<200>	29.2 ± 1.2	24.9 ± 0.6
<311>	27.4 ± 1.3	26.0 ± 1.5
<220>	24.9 ± 1.7	17.5 ± 2.5
<111>	42.8 ± 2.6	—

Rates were determined for plastic strains ranging from 1.5 to 5.0% and have units of MPa per 1% strain

More strikingly, our in situ neutron diffraction studies reveal grain-orientation-dependent work-hardening behaviors that provide insight into the mesoscale mechanism of the initiation of serrations. A polycrystalline alloy, such as IN625, consists of a variety of single-crystal grains oriented differently along the LD. Each subset of the grains is designated here as <*hkl*>//LD where <*hkl*> represents the plane normal of the grains that are aligned in the LD. The effective stress along the LD, $\sigma _{hkl}^{\mathrm{L}}$, acting locally on the <*hkl*>//LD grains, can be approximated by the <*hkl*>-dependent lattice strain in the LD, $\varepsilon _{hkl}^{\mathrm{L}}$, given as $\sigma _{hkl}^{\mathrm{L}} = E_{hkl}^{\mathrm{s}} \cdot \varepsilon _{hkl}^{\mathrm{L}}$^41^, where $E_{hkl}^{\mathrm{s}}$ is the *hkl*-dependent Young’s modulus of the single crystal, which for these samples was reported elsewhere^42^.

**Fig. 5:**
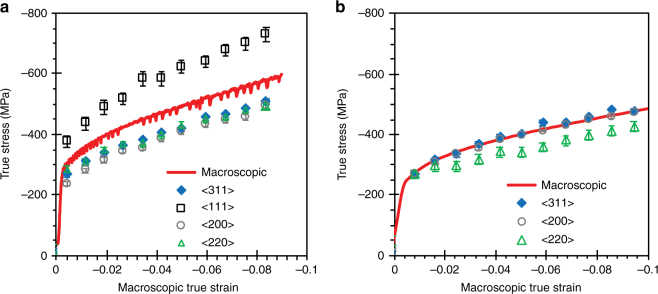
Grain-level stress–strain curves for conventionally processed and additively manufactured Inconel 625. Grain orientation (*hkl*)-dependent effective stress along the loading direction as a function of macroscopic true strain for **a** CP-IN625 and **b** AM-IN625 deformed at 600 °C compared to the respective macroscopic stress–strain curves. Error bars are obtained based on the uncertainties in the lattice strain measurements

The effective work-hardening rate of the <*hkl*>//LD grains can be estimated as $\theta _{hkl} = {\mathrm{d}}\sigma _{hkl}^{\mathrm{L}}/{\mathrm{d}}\varepsilon .$ Figure 5 shows the variation of <*hkl*>-dependent local stresses as a function of true strain compared to the macroscopic true stress–true strain curve for the CP-IN625 and AM-IN625 when deformed at 600 °C, with $\theta _{hkl}$ values listed in Table 1. Notably, the <111>//LD grains in the texture-free, CP-IN625 exhibit the highest work-hardening rate while taking the highest stress among all the <*hkl*>//LD grains throughout loading (Fig. 5a). We postulate that dislocation unlocking may be preferentially initiated in the <111>//LD grains as a result of a lower critical strain being required (i.e., *γ*_c_ is first reached on these grains, see Fig. 4a, b). This causes local stress drops in the <111>//LD grains that propagate into other <*hkl*> grains, eventually leading to plastic instability and macroscopic jerky flow. However, the amount of the <111>//LD grains in the textured AM-IN625 is estimated to be <1%, much smaller than the amount of <100>//LD grains, which is about 86% as estimated from pole figures. In this case, the <100>//LD grains take the majority of the stress as indicated by the fact that the local stress acting on the <100>//LD grains is nearly identical to the macroscopic stress at any plastic strain level (Fig. 5b). As the effective work-hardening rate in the <100> direction is considerably lower than that in the <111> direction, the grain-level strain required to reach the same critical stress for serrations is higher in the <100> direction (see Fig. 4b). Thus the <100>//LD grains are less favored to initiate serrations. As such, on the mesoscopic scale, the lack of <111>//LD grains and the lower sensitivity of the <100>//LD grains to dislocation unlocking would contribute to the disappearance of Type C serrations in AM-IN625, in addition to the sink effect of the secondary phases. Furthermore, the additional studies on heat-treated AM-IN625 indicate that the texture was retained during heat treatments (see Supplementary Fig. 7 for more detail). The slightly muted resulting serrations in the samples with partially or fully dissolved secondary phases provide further evidence that texture of the type present in the AM-IN625 samples suppresses DSA.

## Discussion

In summary, while AM can be used to fabricate complex-shaped components, its adoption for structural applications requires an in-depth understanding of the structure–property relationships in these materials. DSA is undesirable in structural applications as it leads to localized deformation and, in the case of Type C serrations, results in sudden reductions in flow stress with strain. While DSA is active in CP-IN625 at elevated temperatures, it is absent in IN625 made by AM up to 700 °C, pointing to a potential benefit of AM of Ni-base superalloy components that will be used at elevated temperatures. In particular, this is critical as AM would offer the benefit of a route to fabricate complex geometries in this difficult-to-machine alloy, with no undesirable DSA during operation. Additionally, this points to a means for tailoring the microstructure and phases of materials made by AM to avoid undesirable behaviors in operation.

The neutron diffraction studies and the DSA model we propose here offer insights for understanding the effect of dislocation–solute interactions on work-hardening behaviors. This may pave the way for a fundamental understanding of the abnormal increase in mechanical strength at elevated temperatures commonly observed in a wide range of high-temperature structural alloys.

## Methods

### Sample preparation

Powder-based DED with a laser heat source was used to additively manufacture a wall out of pre-alloyed IN625 powder. To deposit the 101 mm long, 7 mm thick, and 28 mm tall IN625 wall onto the CP-IN625 substrate, a laser power of 2 kW, laser travel speed of 10.6 mm s^−1^, layer thickness of 0.9 mm, powder flow rate of 16 g min^−1^, and argon shielding gas flow rate of 9.4 L min^−1^ were used^43^.

Uniaxial compression specimens (5 mm diameter, 10 mm long) were extracted from the AM-IN625 as well as from CP-IN625 plate. The CP-IN625 plate was procured after it was heat treated at 871 °C for 1–4 h, followed by an air quench, in accordance with ASTM B-443 Grade 1^25^. The as-deposited AM-IN625 was not subjected to any heat treatment.

### Neutron diffraction measurements

In situ neutron diffraction characterization of CP-IN625 and AM-IN625 during compression were performed on VULCAN, the engineering diffractometer at the Spallation Neutron Source (SNS), Oak Ridge National Laboratory. Figure 1 shows a top view schematic of the experimental set-up. The red square represents the sampling volume of about 60 mm^3^ defined by the incident beam and the radial collimators. The −90° and +90° detector banks record diffractions from the grains whose lattice planes (*hkl*) are perpendicular to *Q*_1_ and *Q*_2_, respectively, as shown in the two insets. The compression specimen (light-blue rectangle) is positioned at 45° from the incident beam such that Bank 1 probes the strain component along the LD while Bank 2 detects the strain component in the TD simultaneously. Additional test details are provided in Supplementary Note 1.

For high-temperature compression tests, a thermocouple was spot welded onto the center of each sample to monitor its temperature as it was heated using an induction coil. The elevated temperature was held while the sample was compressed using an MTS load frame with alumina platens. Supplementary Fig. 1 is a photograph of the thermomechanical testing set-up at VULCAN. True stresses and true strains during plastic deformation at elevated temperatures were evaluated from measured engineering stresses, together with engineering strains estimated from the relative cross-head displacements after correcting for machine compliance at each temperature.

### Neutron diffraction data reduction and analysis

Under the neutron event-based data acquisition scheme implemented at the SNS, each detected neutron is recorded with a timestamp from a master time clock allowing a versatile and accurate post-experiment data binning. As such, neutron time event data can be easily synchronized with the mechanical loading data during a continuous loading test. The recorded neutron data were reduced using a dedicated software, VDRIVE, and then sliced and binned into histograms corresponding to small temporal intervals that were synchronized with the loading parameters (e.g., force, displacement, temperature). In the present study, each sliced diffraction pattern was obtained by binning the neutron data recorded into a 1-min interval, which was synchronized with the applied force and displacement. Single peak-fitting, as implemented in VDRIVE, was used to extract the information including peak position (*d*-spacing), full-width-at-half-maximum, and integrated intensity for selected *hkl* reflections.

### Data availability

All relevant data are available from the authors.

## Electronic supplementary material

Supplementary Information

## References

[1] Hayes RW (1983). On a proposed theory for the disappearance of serrated flow in f.c.c. Ni alloys. Acta Metall..

[2] Jovanovic M, Djuric B, Drobnjak D (1981). Serrated yielding in commercial Cu-Be-Co alloy. Scr. Metall..

[3] Era H, Ohura N, Onodera R, Shimizu M (1984). Precipitation and the Portevin-Le Chatelier effect in Cu-5.5, 11.6 and 14.2 at.% Ga alloy. Scr. Metall..

[4] Korbel A, Dybiec H (1981). The problem of the negative strain-rate sensitivity of metals under the Portevin-Le Chatelier deformation conditions. Acta Metall..

[5] Wijler A, Schade van Westrum J (1971). Serrated yielding and inhomogeneous deformation in Au (14at%Cu). Scr. Metall..

[6] Robinson JM, Shaw MP (1994). Microstructural and mechanical influences on dynamic strain aging phenomena. Int. Mater. Rev..

[7] Cottrell AH (1953). Dislocation and Plastic Flow in Crystals.

[8] Blakemore JS (1970). The Portevin-Le Chatelier effect in carburized nickel alloys. Metall. Mater. Trans..

[9] Mulford RA, Kocks UF (1979). New observations on the mechanisms of dynamic strain aging and of jerky flow. Acta Metall..

[10] Hayes RW, Hayes WC (1984). A proposed model for the disappearance of serrated flow in two Fe alloys. Acta Metall..

[11] Rodriguez P (1984). Serrated plastic flow. Bull. Mater. Sci..

[12] Kocks UF, Cook RE, Mulford RA (1985). Strain aging and strain hardening in Ni-C alloys. Acta Metall..

[13] Delafosse D, Lapasset G, Kubin LP (1993). Dynamic strain ageing and crack propagation in the 2091 AlLi alloy. Scr. Metall. Mater..

[14] Kishore R, Singh RN, Sinha TK, Kashyap BP (1997). Effect of dynamic strain ageing on the tensile properties of a modified 9Cr – 1Mo steel. J. Mater. Sci..

[15] Srinivasan VS (1997). The influence of dynamic strain ageing on stress response and strain-life relationship in low cycle fatigue of 316L (N) stainless steel. Scr. Mater..

[16] Mannan SL (1993). Role of dynamic strain ageing in low cycle fatigue. Bull. Mater. Sci..

[17] Valsan M, Sastry DH, Rao KB, sankara, Mannan SL (1994). Effect of strain rate on the high-temperature low-cycle fatigue properties of a nimonic PE-16 superalloy. Metall. Mater. Trans. A.

[18] Hörnqvist M, Joseph C, Persson C, Weidow J, Lai H (2014). Dynamic strain aging in Haynes 282 superalloy. MATEC Web Conf..

[19] Kreutz EW, Backes G, Gasser A, Wissenbach K (1995). Rapid prototyping with CO2 laser radiation. Appl. Surf. Sci..

[20] Griffith, M. L. et al. Free form fabrication of metallic components using laser engineered net shaping (LENS^TM^). In *The 7th Solid Freeform Fabrication Symposium* (http://sffsymposium.engr.utexas.edu/Manuscripts/1996/1996-16-Griffith.pdf) 125–132 (Laboratory for Freeform Fabrication and University of Texas at Austin, Austin, TX, 1996).

[21] Keicher DM, Miller WD (1998). LENS^TM^ moves beyond RP to direct fabrication. Met. Powder Rep..

[22] Carroll BE, Palmer TA, Beese AM (2015). Anisotropic tensile behavior of Ti–6Al–4V components fabricated with directed energy deposition additive manufacturing. Acta Mater..

[23] Wang Z, Palmer TA, Beese AM (2016). Effect of processing parameters on microstructure and tensile properties of austenitic stainless steel 304L made by directed energy deposition additive manufacturing. Acta Mater..

[24] Wang XL (2006). VULCAN—the engineering diffractometer at the SNS. Phys. B Condens. Matter.

[25] INCONEL ® alloy 625. Special Metals Corporation, SMC-020 (2006).

[26] Stoica GM, Stoica AD, Miller MK, Ma D (2014). Temperature-dependent elastic anisotropy and mesoscale deformation in a nanostructured ferritic alloy. Nat. Commun..

[27] Vandenbrink SH, van den Beukel A, Mccormick PG (1975). Strain rate sensitivity and Portevin-Le Chatelier effect in Au-Cu alloys. Phys. Status Solidi A Appl. Res..

[28] Dey GK, Albert S, Srivastava D, Sundararaman M, Mukhopadhyay P (1989). Microstructural studies on rapidly solidified Inconel 625. Mater. Sci. Eng. A.

[29] Song KH, Nakata K (2010). Effect of precipitation on post-heat-treated Inconel 625 alloy after friction stir welding. Mater. Des..

[30] Rombouts M, Maes G, Mertens M, Hendrix W (2012). Laser metal deposition of Inconel 625: microstructure and mechanical properties. J. Laser Appl..

[31] Xu F, Lv Y, Liu Y, Xu B, He P (2013). Effect of heat treatment on microstructure and mechanical properties of inconel 625 alloy fabricated by pulsed plasma arc deposition. Phys. Procedia.

[32] Penning P (1972). Mathematics of the portevin-le chatelier effect. Acta Metall..

[33] Gopinath K, Gogia AK, Kamat SV, Ramamurty U (2009). Dynamic strain ageing in Ni-base superalloy 720Li. Acta Mater..

[34] Ramberg, W. & Osgood, W. R. *Description of Stress-Strain Curves by Three Parameters*. National Advisory Committee For Aeronautics Technical Note (National Advisory Committee for Aeronautics, Washington, D.C., 1943).

[35] Sleeswyk AW (1958). Slow strain-hardening of ingot iron. Acta Metall..

[36] Hayes RW, Hayes WC (1982). On the mechanism of delayed discontinuous plastic flow in an age-hardened nickel alloy. Acta Metall..

[37] Ekaputra IMW, Kim WG, Park JY, Kim SJ, Kim ES (2016). Influence of dynamic strain aging on tensile deformation behavior of alloy 617. Nucl. Eng. Technol..

[38] Gale, W. F. & Totemeier, T. C. *Smithells Metals Reference Book* (Butterworth-Heinemann, Burlington, 2004).

[39] Nagesha A (2012). Dynamic strain ageing in Inconel Alloy 783 under tension and low cycle fatigue. Mater. Sci. Eng. A.

[40] Patil RV, Kale GB (1996). Chemical diffusion of niobium in nickel. J. Nucl. Mater..

[41] Ungar T, Stoica AD, Tichy G, Wang XL (2014). Orientation-dependent evolution of the dislocation density in grain populations with different crystallographic orientations relative to the tensile axis in a polycrystalline aggregate of stainless steel. Acta Mater..

[42] Wang Z, Stoica AD, Ma D, Beese AM (2016). Diffraction and single-crystal elastic constants of Inconel 625 at room and elevated temperatures determined by neutron diffraction. Mater. Sci. Eng. A.

[43] Denlinger ER, Heigel JC, Michaleris P, Palmer TA (2015). Effect of inter-layer dwell time on distortion and residual stress in additive manufacturing of titanium and nickel alloys. J. Mater. Process. Technol..

