# Supplementary material for: Absence of dynamic strain aging in an additively manufactured nickel-base superalloy
Source: Nat Commun. 2018 May 25;9:2083. doi: 10.1038/s41467-018-04473-5 (PMC5970170; doi:10.1038/s41467-018-04473-5)
Supplement: Supplementary file 1 — Supplementary Information [file 41467_2018_4473_MOESM1_ESM.pdf]

Supplementary Information:

Absence of dynamic strain aging in an additively manufactured nickel-base superalloy

Beese et al.

Supplementary Note 1: Neutron diffraction characterization

Neutron diffraction setup

A picture of a compression specimen installed in the VULCAN test frame is given in Supplementary Fig. 1.

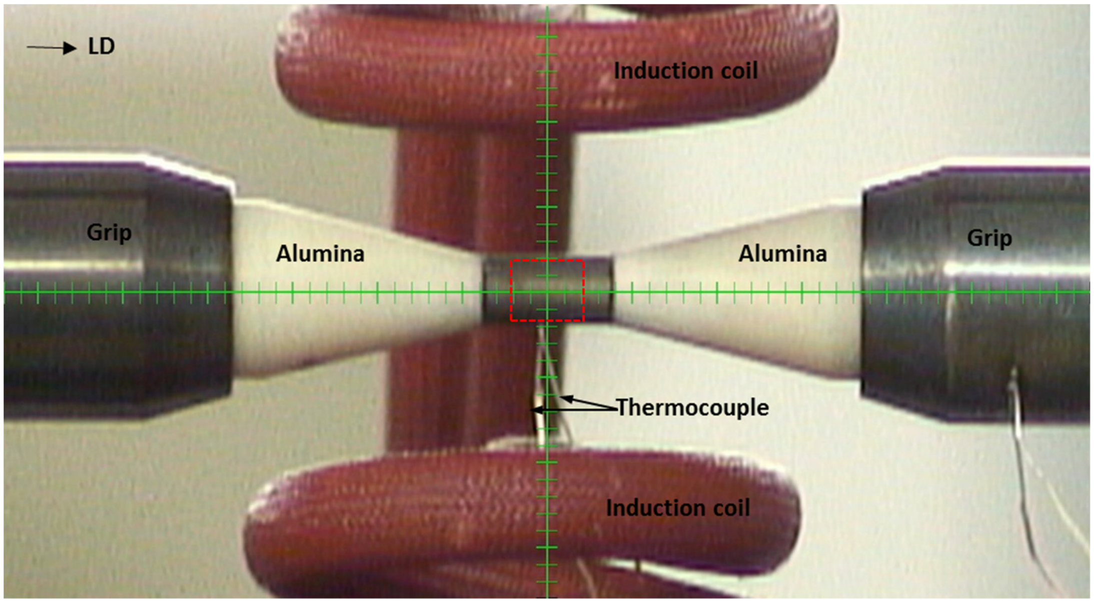

Supplementary Figure 1. Picture (perpendicular to the loading axis) showing *in situ* neutron diffraction measurement during compressive loading of an Inconel 625 (IN625) specimen (10 mm gauge length shown) at an elevated temperature. The red dashed rectangle denotes where the incident neutron beam shines. Here, LD denotes the loading direction.

Neutron diffraction characterization of texture

Texture measurements of an additively manufactured IN625 wall were conducted on the VULCAN beamline, where rotations around the vertical and horizontal axes were implemented by the sample stage and a goniometer, respectively. Supplementary Fig. 2 shows the (200), (220), and (111) pole figures indicative of a strong Goss texture component {011} <001>, in which the normal of the (011) plane is oriented along the build vertical direction (BVD, center of the pole

figures) and the [001] direction inside the (011) plane is along the build length direction (BLD). Since the cylindrical compression samples were cut along the build length direction, the <001> grains were preferentially aligned along the compression axis, resulting in a strong <001> texture along the loading direction (LD).

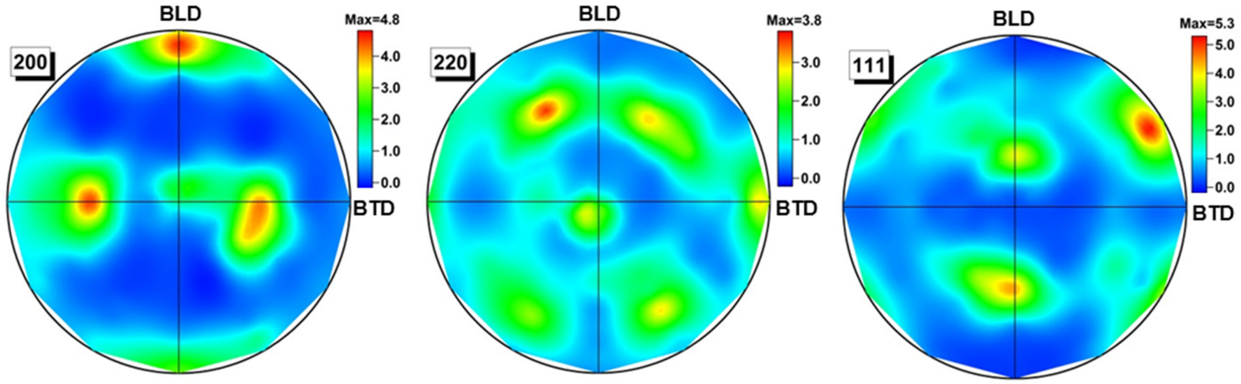

Supplementary Figure 2. (200), (220), and (111) pole figures of the AM build determined from neutron diffraction, indicative of a Goss texture component {011} <001>. Here, BTD denotes the thickness direction of the build.

Supplementary Note 2: Mechanical tests

Additional compression tests at room temperature and 600-700 °C

In addition to the compression tests performed at 600 ^{o}C, compression tests were performed on conventionally processed IN625 (CP-IN625) and as-deposited additively manufactured IN625 (AM-IN625) at room temperature (RT) and at 700 °C, with test parameters listed in Supplementary Table 1. At room temperature, the stress-strain curves were smooth as shown in Supplementary Fig. 3. At 700 °C, the AM-IN625 still did not exhibit any serrations in the stress-strain curve, but the CP-IN625 did exhibit serrations indicative of dynamic strain aging as shown in Supplementary Fig. 4.

Supplementary Table 1. Test parameters for compression experiments.

**Temperature (^{o}C)**	RT		600		700	
**Processing method**	CP	AM	CP	AM	CP	AM
**Heating rate (^{o}C s^{-1})**	-	-	0.5	0.5	0.6	0.6
**Soak time at temperature prior to compressive loading (min)**	-	-	5	5	5	5
**Strain rate (s^{-1})**	2.0×10^{-5}		$1.5 \times 10^{-5} – 1.9 \times 10^{-4}$		1.5×10^{-5}	

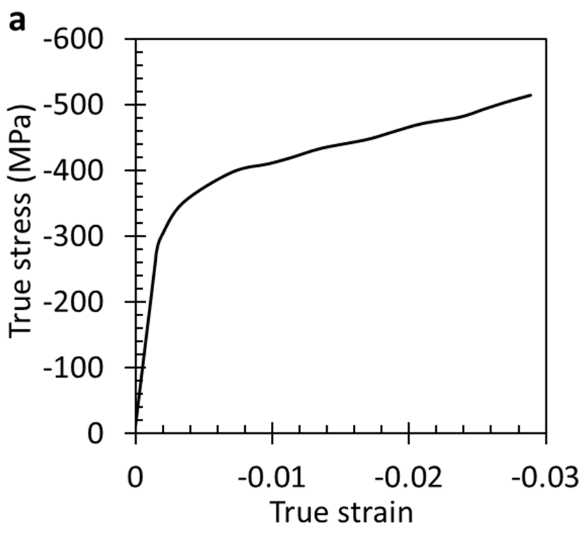

Supplementary Figure 3. Macroscopic true stress-strain curves for **a** CP-IN625 and **b** AM-IN625 samples deformed at room temperature.

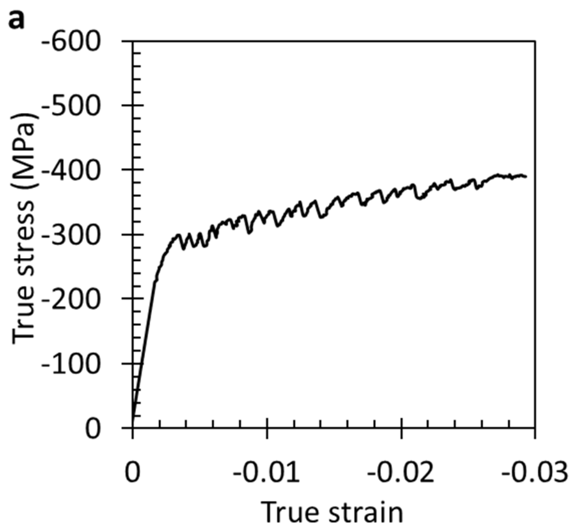

Supplementary Figure 4. Macroscopic true stress-strain curves for **a** CP-IN625 and **b** AM-IN625 samples deformed at 700 ºC.

Additional compression tests on heat treated samples

To elucidate the impact of the secondary phases in the additively manufactured material, we heat treated the additively manufactured samples in a vacuum furnace under the following three conditions (followed by furnace cooling): (1) 980°C for 1 hour (Samples D and E), (2) 1095°C for 1 hour (Sample F), and 1200°C for 10 hours (Samples G, H, and I), as summarized in Supplementary Table 2. Elevated temperature compression tests were performed on these samples, as well as AM-IN625 and CP-IN625 without heat treatment (Samples A-C), without neutron diffraction.

Secondary phases were barely dissolved in Samples D-F, and these samples, like as-deposited AM-IN625, exhibited no Type-C serrations in the stress-time (or strain) curves when tested at 600°C, 650°C, or 700°C at a strain rate of ~2.5×10^{-5} s^{-1}.

In contrast, secondary phases were found to be partially (with an area fraction of 1.0%, compared to an area fraction of 1.7% in as-deposited AM-IN625) dissolved in Samples G and H. When these two samples were tested at 680°C and 700°C, they showed Type-C like serrations with a stress-drop value as much as 1/3 to 1/2 of that for typical Type-C serrations observed in CP-IN625 (see Supplementary Fig. 5). This shows that partial dissolution of secondary phases led to the occurrence of Type-C like serrations.

Despite the same nominal heat treatments, secondary phases were found to be nearly fully dissolved in Sample I, but not in Samples G or H. Sample I was heat treated under a vacuum level of ~$2.0\text{x}10^{-5}$ Torr, while the vacuum sensor was not functional during the heat treatment of Samples G and H, and therefore, it is believed that the vacuum level did not reach the set value of $2.0\text{x}10^{-5}$ Torr. Prior work has shown that oxygen contributes to carbide precipitation[1], therefore, the lower oxygen level in the furnace due to insufficient vacuum during the heat treatment of

Sample I may explain the nearly full dissolution of secondary phases in that sample, with only a partial dissolution of secondary phase in Samples G and H.

Sample I was tested at 600°C up to a plastic strain of 3%, and then subjected to a subsequent test at 680°C, at a strain rate of ~2.5×10^{-5} s^{-1}. No Type-C serrations were observed during the 600°C test, while one prominent serration with a slightly lower stress drop than observed in CP-IN625 (Sample B) was discernible in the subsequent 680°C test, as shown in Supplementary Fig. 6.

Pole figures of as-deposited AM-IN625 and compressed Samples G and I were measured using neutron diffraction (VULCAN instrument at ORNL), as shown in Supplementary Fig. 7. The as-deposited Goss texture was largely retained after heat treatment and compression tests. The retained texture, as well as the undissolved secondary phases, may explain why the observed Type-C like serrations in G and H exhibited a much lower stress drop (~6 MPa) compared to that typically observed in CP-IN625 (~15 MPa). Similarly, the absence of DSA in Sample I when tested at a temperature as low as 600°C may also be due to the suppression effect of the texture retained after heat treatment.

It is evident that DSA occurred in AM-IN625 after heat treatment at sufficiently high temperatures that resulted in either partial or complete dissolution of the secondary phases. These findings provide added support for our conclusion that the absence of DSA in additively manufactured IN625 is indeed due to finely-dispersed secondary phases and textured grains.

Supplementary Table 2. Summary of heat-treated AM-IN625 samples and compression test results [a].

Sample ID	Material	Heat treatment	Carbides or secondary phases	Test temperature	Type-C serration
**A**	AM-IN625	No	As deposited	1st test at 600°C<br>2nd test at 680°C	Absent<br>Absent
**B**	CP-IN625	No	As received	1st test at 600°C<br>2nd test at 680°C	Present<br>Present
**C**	CP-IN625	No	As received	650°C	Present
**D**	AM-IN625	**HT980C**: Heat-treated at 980°C for 1 hr	Barely dissolved	600°C	Absent
**E**	AM-IN625	**HT980C**: Heat-treated at 980°C for 1 hr	Barely dissolved	700°C	Absent
**F**	AM-IN625	**HT1095C**: Heat-treated at 1095°C for 1 hr	Barely dissolved	650°C	Absent
**G**	AM-IN625	**HT1200C**: Heat-treated at 1200°C for 10 hrs	Partially dissolved	680°C	Present with low stress-drop
**H**	AM-IN625	**HT1200C**: Heat-treated at 1200°C for 10 hrs	Partially dissolved	700°C	Present with low stress-drop
**I**[b]	AM-IN625	**HT1200C**: Heat-treated at 1200°C for 10 hrs	Fully dissolved	1st test at 600°C<br>2nd test at 680°C	Absent<br>Present

[a] *A strain rate of* $2.5x10^{-5}$ s^{-1} *was used for all the tests.*

[b] *Although using same parameters, Sample I was heat treated separately from Samples G and H with vacuum set to* $2.0x10^{-5}$ *Torr in Sample I, and not monitored in G or H.*

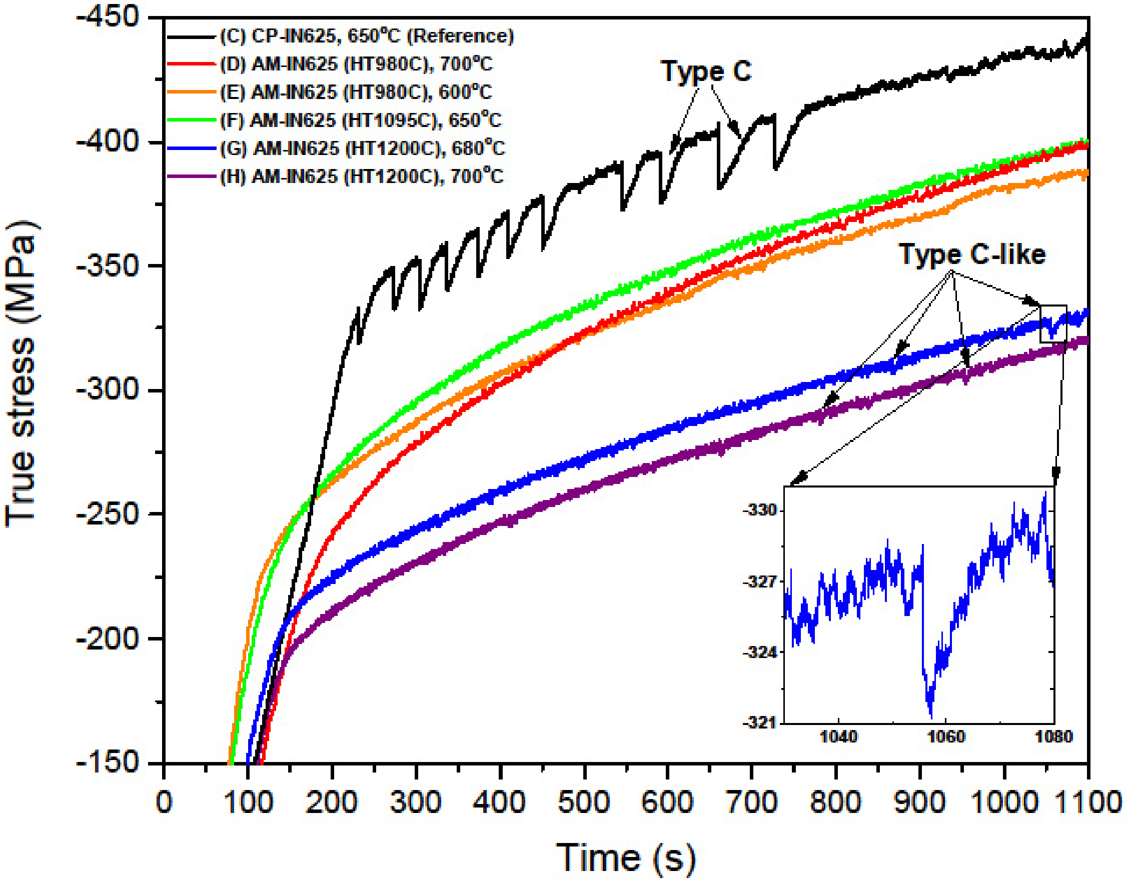

Supplementary Figure 5. True stress-time curves determined for heat-treated AM-IN625 samples (D-H), compared to that of CP-IN625 (Sample C), which had typical Type C serrations.

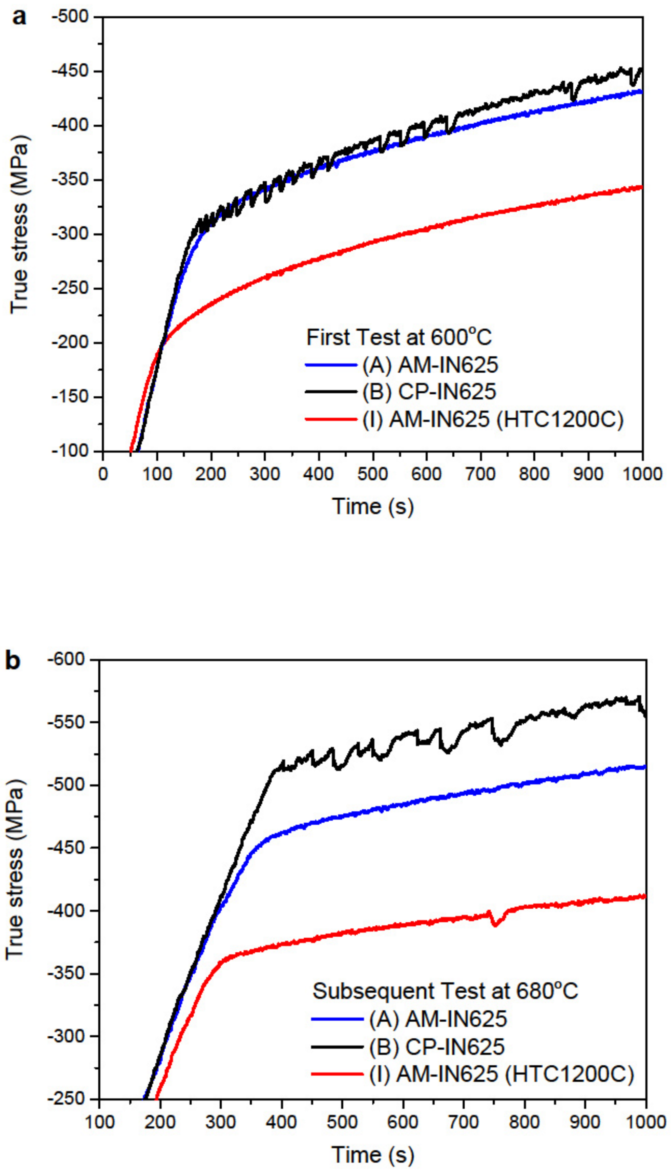

Supplementary Figure 6. True stress-time curves of Sample I (AM-IN625 annealed at 1200°C for 10 hrs) determined from compression tests **a** first at 600°C and **b** subsequently at 680°C. For comparison, also shown are those of Sample A (as-deposited AM-IN625) and Sample B (as-received CP-IN625) tested identically.

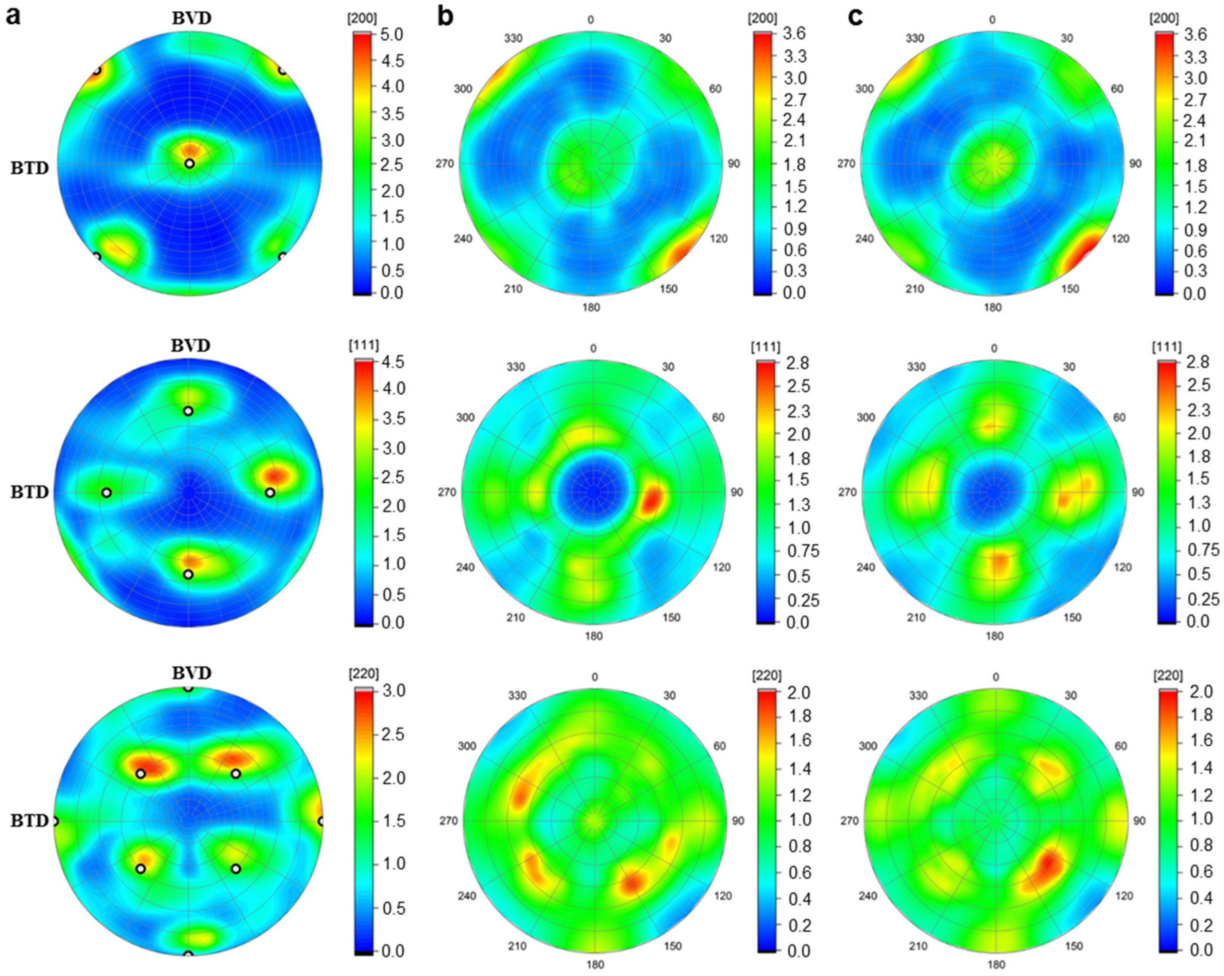

Supplementary Figure 7. Neutron diffraction determined (200), (111) and (220) pole figures for **a** as-deposited AM-IN625, and AM-IN625 samples heat treated at 1200ºC with **b** fully dissolved secondary phases (Sample I), and **c** partially dissolved secondary phases (Sample G). BVD is the build vertical direction, BTD is the build thickness direction, and the center of the pole figures is the build length direction.

Supplementary Note 3: Microstructural characterization

Scanning Electron Microscopy and Electron Backscatter Diffraction

For microstructural evaluation, the samples were polished using standard metallographic procedures, followed by electrolytic etching using 10 wt.% oxalic acid in DI water. The CP-IN625 and AM-IN625 samples were examined in a scanning electron microscope (SEM, FEI Quanta 200), as shown in Supplementary Fig. 8. The average size of the carbides in the CP-IN625 was found to be 3.1 μm^2, occupying an area fraction of 0.9 %. The average size of the secondary phases in the AM-IN625 was found to be 2.2 μm^2, occupying an area fraction of 1.7 %.

The chemical compositions of the CP-IN625 and AM-IN625 samples were evaluated using inert gas fusion (C and N) and inductively coupled plasma optical emission spectroscopy (ICP-OES for the remaining elements), with the results shown in Supplementary Table 3.

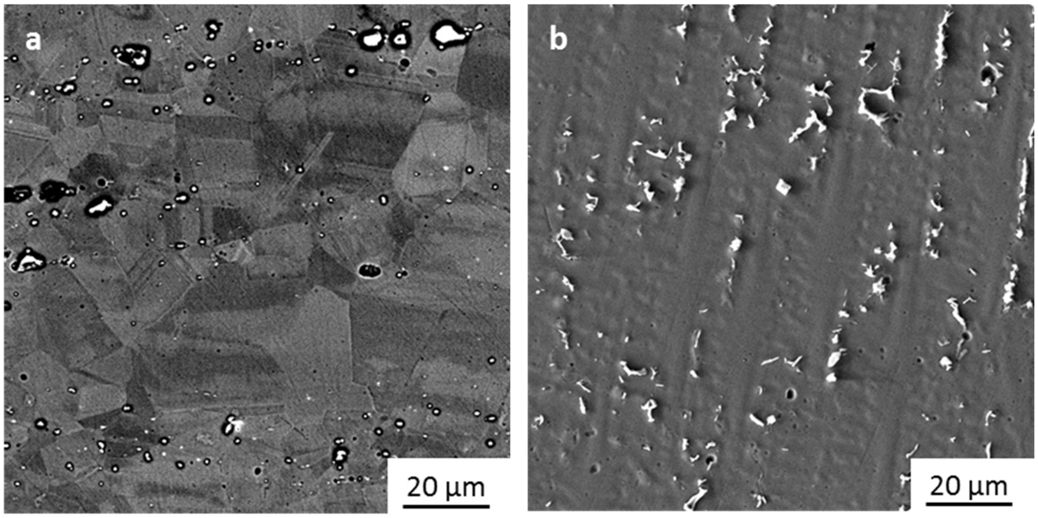

Supplementary Figure 8. a Back-scattered electron image of etched CP-IN625 with Mo- and Ni-rich (bright), and Ti-rich (dark) carbides. **b** Secondary electron image of etched AM-IN625 with Mo- and Nb-rich carbides, N-rich phase, and Laves phase. The normal of the cross-section is parallel to the loading direction of the sample.

Supplementary Table 3. Elemental composition (wt.%) of CP-IN625 and AM-IN625 compared with manufacturer specifications for IN625[2].

	**Manufacturer**	**CP-IN625**	**AM-IN625**	**Uncertainty**
Cr	21.5	21.4	22.5	0.2
Mo	8.75	8.77	8.55	0.09
Fe	4.58	4.65	4.44	0.04
Nb+Ta	3.49	3.49	3.35	0.01
Co	0.28	0.33	<0.01	0.01
Mn	0.26	0.27	0.35	0.01
Si	0.25	0.20	0.32	0.01
Ti	0.24	0.23	0.01	0.01
Al	0.19	0.01	<0.01	0.01
C	0.027	0.012	0.020	0.001
P	<0.005	0.009	0.005	0.001
S	<0.002	0.003	0.001	0.001
N	-	0.091	0.039	0.006
Cu	-	0.066	0.022	0.001

Additionally, EBSD was used to determine the grain size and shape in CP-IN625 and in cross-sections normal to length (Fig. 3c), thickness, and vertical directions in AM-IN625 as shown in Supplementary Fig. 9. In order to describe the grain shape, the grains can be approximated as elliptical, such that shape is described by their aspect ratio, or the ratio of major to minor axis. An aspect ratio of 1 corresponds to an equiaxed grain, and larger values correspond to elongated grains.

A summary of the grain size and morphology is given in Supplementary Table 4. Compared with the CP-IN625, the AM-IN625 had larger and more elongated grains with a wider distributions of grain size and aspect ratio in all three orientations. The grain size affects the yield strength through grain boundary strengthening, which will indirectly influence the critical strain rate for DSA to occur.

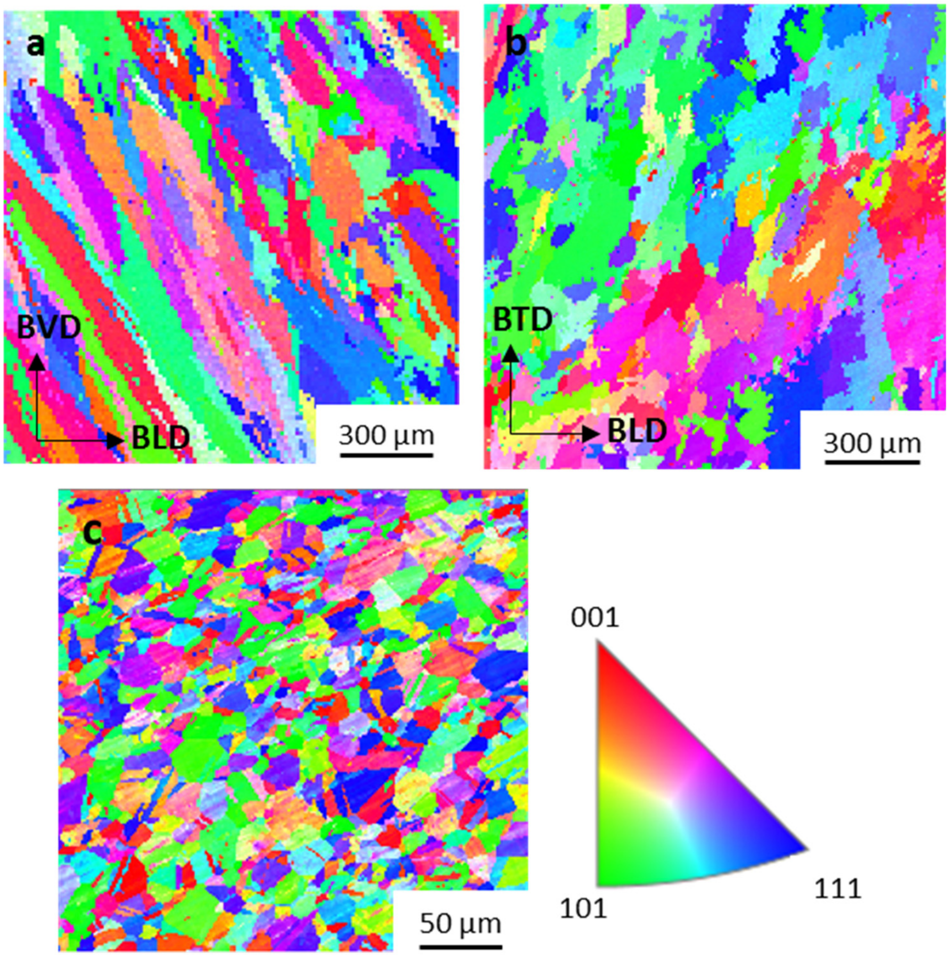

Supplementary Figure 9. EBSD maps of **a** the cross-section whose normal is parallel to the build thickness direction (BTD) and **b** the cross-section whose normal is parallel to the build vertical direction (BVD) in AM-IN625, and **c** CP-IN625. Here, BLD denotes the build length direction. In all figures, the colors correspond to the *hkl* plane normals coming out of the page.

Supplementary Table 4. Grain areas and aspect ratios in CP-IN625 and AM-IN625.

	**Mean grain area (μm^2)**	**Mean grain aspect ratio**
**CP**	400	1.8
**AM, normal to thickness**	16500	4.3
**AM, normal to vertical direction**	8500	2.5
**AM, normal to length**	19700	4.2

Transmission Electron Microscopy

Transmission electron microscopy (TEM) studies showed that the AM-IN625 sample contained Laves phase, MC with N interstitials or M_xCN, and $(Cr,Nb)_2N$ in addition to the γ matrix, as described below. A thin foil was extracted from AM-IN625 using focused ion beam (FIB; FEI Helios Nanolab 660) milling, and examined using TEM (FEI Talos F200X). Supplementary Fig. 10 is a high-angle annular dark-field (HAADF) image of a Nb-rich area, denoted as Region 1, with four possibly disparate phases denoted as Objects 1 and 2, 3, 4, and 5. EDS maps of the major elements in this region are given in Supplementary Fig. 11, with the elemental compositions of these objects given in Supplementary Table 5. Objects 1, 2, and 4 were identified to be Laves phase[3]. Object 3 was rich in Nb, Mo, and N, and depleted in Ni and Fe, and determined to be $(Cr, Nb)_2N$ with Cr and Nb interstitials. Object 5 was the γ matrix, and the at.% of elements match well with the specifications of the γ phase in IN625.

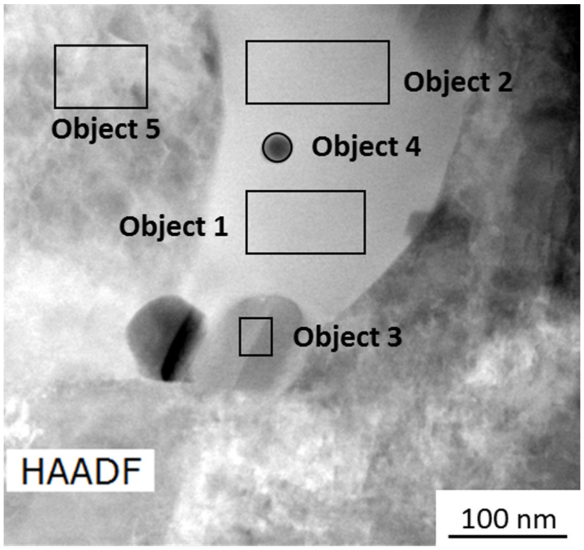

Supplementary Figure 10. TEM HAADF image of Nb-rich Region 1 from a FIB foil extracted from AM-IN625.

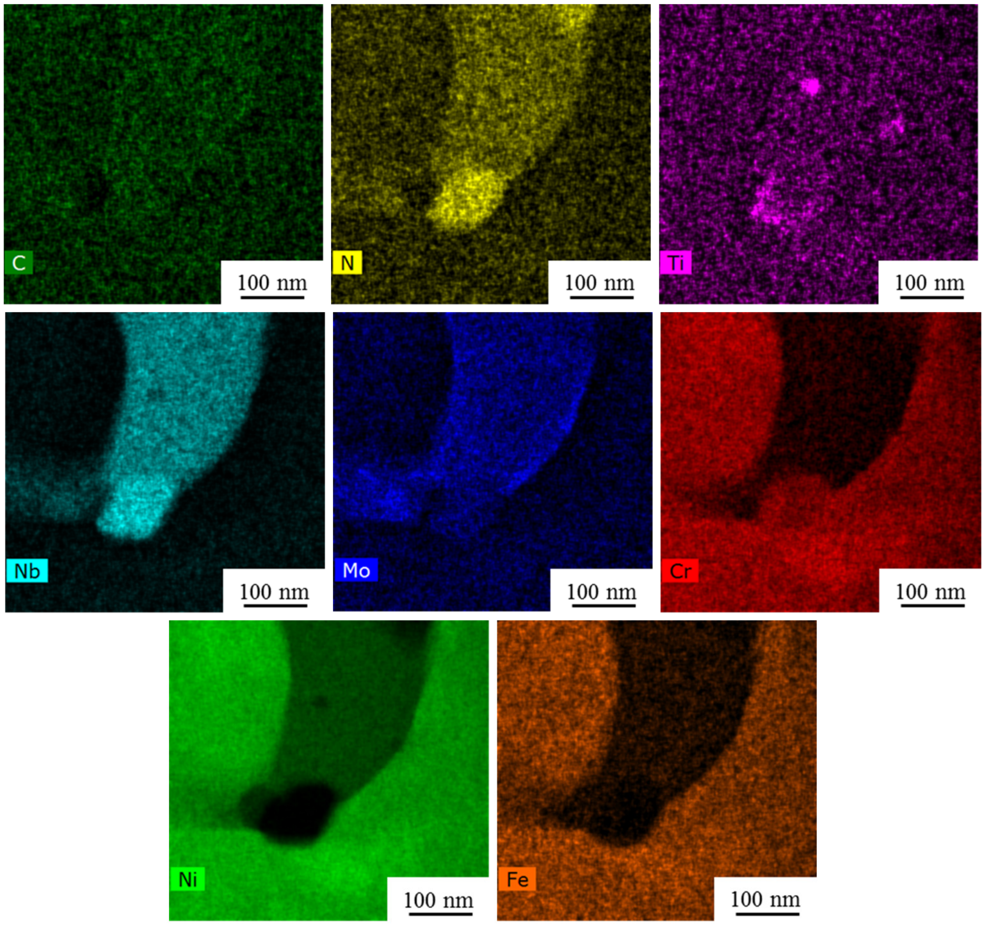

Supplementary Figure 11. EDS maps of a region in AM-IN625 (Region 1 in Supplementary Fig. 10) showing the distribution of major elements.

Supplementary Table 5. Elemental composition (at. %) within AM-IN625 (Objects 1-5 from Region 1 in Supplementary Fig. 10 and Object 6 from Region 2 in Supplementary Fig. 12) compared to the manufacturer specified composition of IN625.

	Ni	Cr	Fe	Nb	Mo	Ti	N	C	Si	O	Co	Mn	Al	(Fe+Cr+Ni) /(Nb+Mo+Ti)	Phase identity
Obj. 1	34.0	16.5	1.6	24.6	11.8	0.04	4.8	1.2	3.8	1.4	0.2	0.08	0	1.43	Laves
Obj. 2	34.8	17.3	1.7	25.2	11.4	0.05	4.0	0.9	3.3	1.1	0.1	0	0.1	1.47	Laves
Obj. 3	1.9	28.9	0.3	42.4	13.1	0.2	12.7	0	0.3	0	0	0.2	0	0.56	$(Cr,Nb)_2N$
Obj. 4	23.9	21.7	1.2	18.1	8.2	6.9	3.1	0	2.7	8.4	0.1	4.5	1.3	1.41	Laves
Obj. 5	62.3	23.7	4.7	2.1	4.6	0.02	0	0.1	0.6	1.4	0.1	0.3	0.1	13.50	γ
Obj. 6	2.0	25.8	0.6	36.0	10.3	0.4	20.0	1.6	0.5	2.7	0	0.03	0.1	0.61	M_xCN
IN625 γ [2]	60.7	24.8	4.9	2.2	5.5	0.3	0	0.1	0.5	0	0.3	0.3	0.4	11.30	

Supplementary Fig. 12a shows another Nb-rich region extracted from AM-IN625, denoted as Region 2. EDS maps of the particle denoted as Object 6 (not shown) indicate it is also rich in Nb, Mo, and N, and depleted in Ni and Fe. The elemental composition of this particle (see Supplementary Table 5) show that it is rich in C compared to the matrix. High resolution images of the particle were taken, and fast Fourier transform (FFT) was used to covert these to diffraction patterns, with a representative pattern given in Supplementary Fig. 12b. By combining the information from the diffraction pattern (Supplementary Fig. 12b) and EDS, Object 6 was identified to be MC with N interstitials or M_xCN, where x = 1, 2.

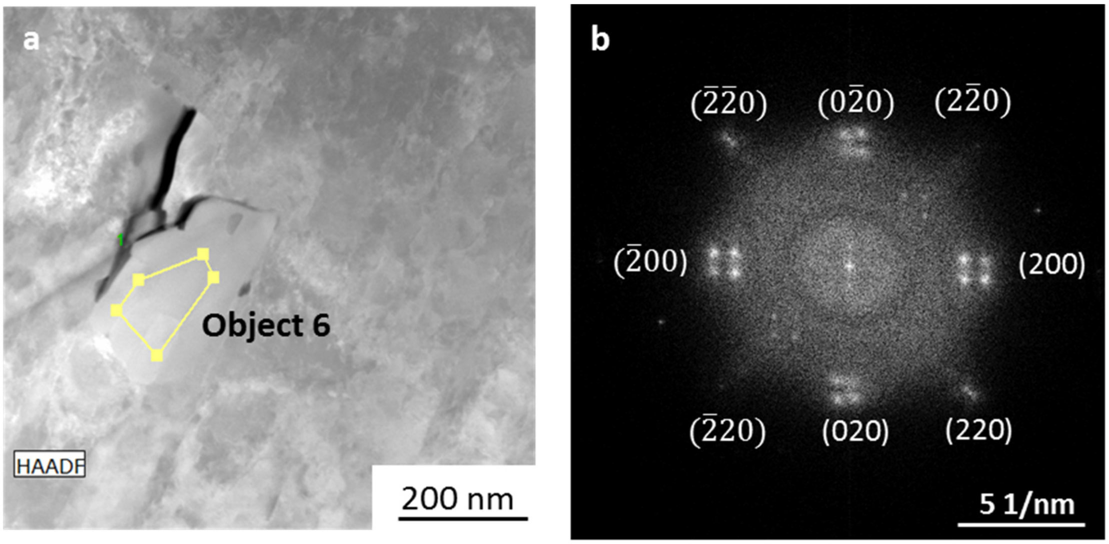

Supplementary Figure 12. **a** TEM HAADF image of Nb-rich Region 2 from a FIB foil extracted from AM-IN625. **b** Indexed diffraction pattern of the particle, denoted as Object 6, in **a** based on an MC crystal structure.

X-ray diffraction

X-ray diffraction (XRD) with Cu K-α radiation was also performed on AM-IN625. The peaks of carbides are located between 35° and 44° [4], as shown in Supplementary Fig. 13, which

indicates the presence of M_6C and MC at 35.6°–36.0 °, $M_{23}C_6$ at 37.8°–38.1°, M_6C at 39.7°, and MC and $M_{23}C_6$ at 41.4°–41.9°, consistent with [4]. A summary of the possible phases identified by literature and the XRD database in JADE software[5] is given in Supplementary Table 6. Our XRD results indicate that the secondary phases present in AM-IN625 are carbides, Laves phase, and N-rich phases (possibly CrNbN and M_2CN).

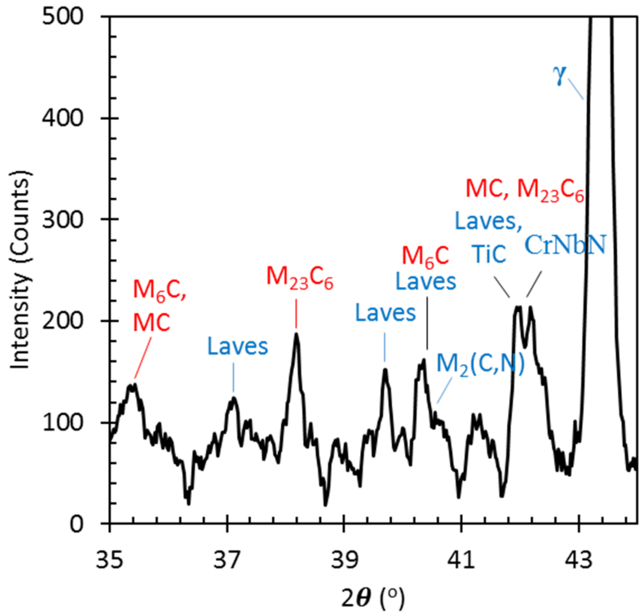

Supplementary Figure 13. XRD spectrum of AM-IN625 used to identify carbides. Phases in blue were identified by JADE software[5], and phases in red were identified by comparison with [4].

Supplementary Table 6. XRD peak positions of secondary phases in IN625 with Cu K-α radiation.

Peak position (2θ, º)	Secondary phases	
	XRD database[5]	Literature[4]
35.6-36.0	-	M_6C, MC
37.1	Laves	-
37.8-38.1	-	$M_{23}C_6$
39.7	Laves	M_6C
40.2	Laves	-
40.6	M_2CN	-
41.9	Laves, TiC	-
41.4-42.0	-	MC, $M_{23}C_6$
42.2	CrNbN	-

Summary of microstructures in CP-IN625 and AM-IN625

A summary of the microstructures identified in the CP-IN625 and AM-IN625 is given in Supplementary Table 7.

Supplementary Table 7. Summary of microstructures in AM-IN625 and CP-IN625.

<table>
<tr><th colspan="2" rowspan="2">Microstructure</th><th colspan="4">AM-IN625</th><th colspan="4">CP-IN625</th></tr>
<tr><th colspan="3">Observations</th><th>Evidence</th><th colspan="3">Observations</th><th>Evidence</th></tr>
<tr><td>Matrix</td><td>Phase identity</td><td colspan="3">FCC γ-Ni</td><td>Neutron diffraction</td><td colspan="3">FCC γ-Ni</td><td>Neutron diffraction</td></tr>
<tr><td></td><td></td><td colspan="3"></td><td></td><td colspan="3"></td><td></td></tr>
<tr><td rowspan="6">Precipitate</td><td>Phase identity</td><td colspan="3">Carbides: MC, M_6C, $M_{23}C_6$;
Laves phases;
N-rich phases: possibly CrNbN and M_2CN</td><td>Ref.[3,4,6]
TEM & XRD</td><td colspan="3">Carbides: primarily (Nb,Ti)C, Mo_6C, $Cr_{23}C_6$, and $Nb_{23}C_6$</td><td>Ref.[7,8]</td></tr>
<tr><td>Morphology</td><td colspan="3">Laves: rod shape
Carbides: cuboidal shape</td><td>Ref.[3,6]
TEM</td><td colspan="3">M_6C: rod shape
MC and $M_{23}C_6$: cuboidal shape</td><td>Ref.[7,8]</td></tr>
<tr><td>Size or distribution</td><td colspan="3">Carbides: diagonal length ~0.3-1 μm
Laves: ~1.1 μm long
N-rich phase: diagonal length ~0.1-0.3 μm</td><td>Ref.[3,6]
TEM</td><td colspan="3">MC and $M_{23}C_{6:}$ diagonal length ~ 0.2 μm
M_6C: ~ 0.3 μm long</td><td>Ref.[7,8]</td></tr>
<tr><td>Volume fraction</td><td colspan="3">~1.7%</td><td>SEM & EBSD</td><td colspan="3">~0.9%</td><td>SEM & EBSD</td></tr>
<tr><td>Interparticle spacing</td><td colspan="3">finer, ~4-10 μm</td><td>Fig. 3b</td><td colspan="3">coarser, ~21-50 μm</td><td>Fig. 3a</td></tr>
<tr><td>Location</td><td colspan="3">Primarily in grain interiors and along grain boundaries</td><td>SEM & EBSD</td><td colspan="3">Primarily along grain boundaries</td><td>SEM & EBSD</td></tr>
<tr><td></td><td></td><td colspan="3"></td><td></td><td colspan="3"></td><td></td></tr>
<tr><td rowspan="4">Grain</td><td></td><td>Average size (μm^2)</td><td>Shape/ average aspect ratio</td><td>Texture</td><td></td><td>Average size (μm^2)</td><td>Shape/ average aspect ratio</td><td>Texture</td><td></td></tr>
<tr><td>Normal to length direction</td><td>19700</td><td>Columnar shape/4.2</td><td>Goss texture</td><td>Fig. 3c</td><td rowspan="3">400</td><td rowspan="3">Close to equiaxed grains/ 1.8</td><td rowspan="3">Texture-free or isotropic</td><td rowspan="3">Supp. Fig. 9c</td></tr>
<tr><td>Normal to thickness direction</td><td>16500</td><td>Columnar shape/4.3</td><td>Goss texture</td><td>Supp. Fig. 9a</td></tr>
<tr><td>Normal to vertical height direction</td><td>8500</td><td>Columnar shape/2.5</td><td>Goss texture</td><td>Supp. Fig. 9b</td></tr>
</table>

Supplementary Note 4: Activation energy calculation

Previous research on DSA has used the critical strain, ε_c, which is the plastic strain for the onset of serrations, as a function of strain rate, $\dot{\varepsilon}$, and temperature, T, to estimate the activation energy of DSA, which is indicative of the underlying dominant mechanism[9,10]. The activation energy, Q, can be calculated through the following relationship[11]:

$$\varepsilon_c^{m+\beta} = K\dot{\varepsilon}\exp\left(\frac{Q}{RT}\right) \tag{1}$$

where m describes the relation between the vacancy concentration and plastic strain, β describes the relation between mobile dislocation density and plastic strain, K is a material constant, and R is the universal gas constant. By fitting Supplementary Eq. (1) to the values of ε_c determined from stress-strain curves of CP-IN625, with temperatures from 550 °C to 650 °C, and with strain rates from 1.0×10^{-5} s^{-1} to 9.5×10^{-4} s^{-1}, as shown in Supplementary Fig. 14, we determined that $m+\beta = 9.6$, $Q = 121$ kJ mol^{-1}, and $K = 8.0\times10^{-29}$.

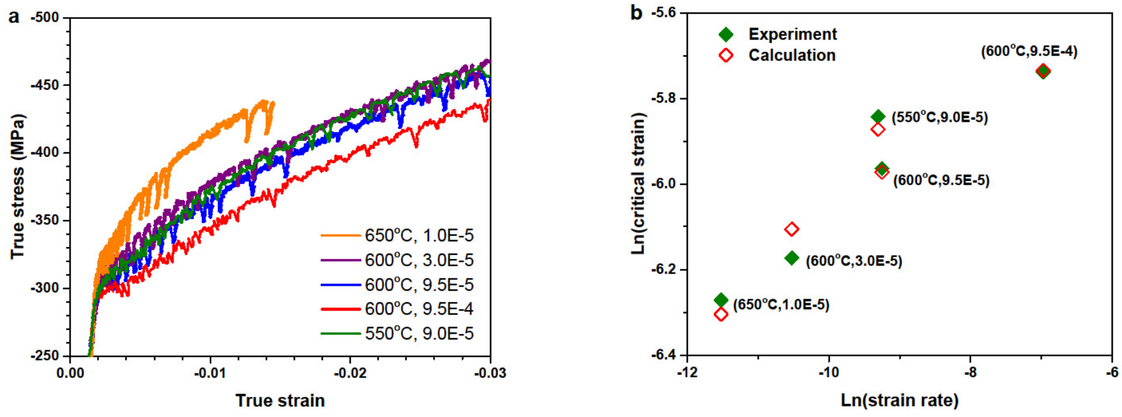

Supplementary Figure 14. a True stress-true strain curves for CP-IN625 at various temperatures and strain rates. **b** Critical strains, determined from **a** (green diamonds), were fitted using Supplementary Eq. (1) to determine the apparent activation energy (Q), where open red diamonds represent the calculated critical strain values based on the best fit parameters.

In previous studies on DSA in Ni-base superalloys where it was concluded that C was responsible for the serrations, the activation energies were found to be in the range of 48-130 kJ mol^{-1} [12–18]. This value is typically compared with the activation energy for diffusion of C in Ni, which is 138 kJ mol^{-1} for lattice diffusion and 69 kJ mol^{-1} for pipe diffusion[19]. The activation energies for substitutional atom diffusion in Ni-base superalloys are in the range of 170-260 kJ mol^{-1} [14–16]. For example, the activation energy for diffusion of Cr in Inconel 783 is 171-184 kJ mol^{-1} [15] and that for diffusion of Nb in Ni-base superalloys is about 202 kJ mol^{-1} [20]. The computed activation energy in this study of 121 kJ mol^{-1} is closer to the activation energy for C diffusion than substitutional atom diffusion in Ni-base superalloys, indicating that the DSA observed under the presently studied temperature and strain rate conditions was primarily controlled by interstitial C.

In addition, as shown in Supplementary Table 3, there is little to no difference in the concentration of substitutional elements (Mo, Nb, and Cr) in the conventionally processed versus additively manufactured IN625. Therefore, the interstitial elements appear to be responsible for the disparate behavior in the AM and CP forms of the same alloy.

Supplementary References

1. Liu, H. S. *et al.* Effect of oxygen content and heat treatment on carbide precipitation behavior in PM Ni-base superalloys. *Int. J. Miner. Metall. Mater.* **19,** 827–835 (2012).
2. INCONEL ® alloy 625. *Special Metals Corporation, SMC-020* (2006).
3. Xu, F. *et al.* Microstructural Evolution and Mechanical Properties of Inconel 625 Alloy during Pulsed Plasma Arc Deposition Process. *J. Mater. Sci. Technol.* **29,** 480–488 (2013).
4. Rombouts, M., Maes, G., Mertens, M. & Hendrix, W. Laser metal deposition of Inconel 625: Microstructure and mechanical properties. *J. Laser Appl.* **24,** 1–6 (2012).
5. Computer software JADE. *Materials Data, Inc* (2010).
6. Silva, C. C. *et al.* New insight on the solidification path of an alloy 625 weld overlay. *J. Mater. Res. Technol.* **2,** 228–237 (2013).
7. Ferrer, L., Pieraggi, B. & Uginet, J. F. Microstructural Evolution During Thermomechanical Processing of Alloy 625. in *Superalloys 718,625 and Various Derivatives* 217–228 (1991).
8. Floreen, S., Fuchs, G. E. & Yang, W. J. The Metallurgy of Alloy 625. in *Superalloys 718, 625, 706 and Various Derivatives. The Minerals, Metals & Materials Society* 13–37 (1994). doi:1
9. Chatterjee, A., Sharma, G., Tewari, R. & Chakravartty, J. K. Investigation of the Dynamic Strain Aging and Mechanical Properties in Alloy-625 with Different Microstructures. *Metall. Mater. Trans. A Phys. Metall. Mater. Sci.* **46A,** 1097–1107 (2015).
10. Shankar, V., Valsan, M., Bhanu Sankara Rao, K. & Mannan, S. L. Effects of Temperature and Strain Rate on Tensile Properties and Activation Energy for Dynamica Strain Aging in Alloy 625. *Metall. Mater. Trans. A* **35,** 3129–3139 (2004).
11. Mccormick, P. G. A model for the Portevin-Le Chatelier effect in the substitutional alloys. *Acta Metall.* **20,** 351–354 (1972).
12. Hörnqvist, M., Joseph, C., Persson, C., Weidow, J. & Lai, H. Dynamic strain aging in Haynes 282 superalloy. in *MATEC Web of Conferences* **14,** 1–6 (2014).
13. Gopinath, K., Gogia, A. K., Kamat, S. V. & Ramamurty, U. Dynamic strain ageing in Ni-base superalloy 720Li. *Acta Mater.* **57,** 1243–1253 (2009).
14. Hale, C. L., Rollings, W. S. & Weaver, M. L. Activation energy calculations for

discontinuous yielding in Inconel 718SPF. *Mater. Sci. Eng. A* **300,** 153–164 (2001).

15. Nagesha, A. *et al.* Dynamic strain ageing in Inconel Alloy 783 under tension and low cycle fatigue. *Mater. Sci. Eng. A* **546,** 34–39 (2012).
16. Nalawade, S. A., Sundararaman, M., Kishore, R. & Shah, J. G. The influence of aging on the serrated yielding phenomena in a nickel-base superalloy. *Scr. Mater.* **59,** 991–994 (2008).
17. Nakada, Y. & Keh, A. S. Serrated flow in Ni-C alloys. *Acta Metall.* **18,** 437–443 (1970).
18. Hayes, R. W. & Hayes, W. C. On the mechanism of delayed discontinuous plastic flow in an age-hardened nickel alloy. *Acta Metall.* **30,** 1295–1301 (1982).
19. Gale, W. F. & Totemeier, T. C. *Smithells Metals Reference Book*. (2004).
20. Patil, R. V. & Kale, G. B. Chemical diffusion of niobium in nickel. *J. Nucl. Mater.* **230,** 57–60 (1996).
